# Subjective effects of broadband water sounds with inaudible high-frequency components

**DOI:** 10.1038/s41598-024-57749-w

**Published:** 2024-04-01

**Authors:** Takumi Asakura

**Affiliations:** Department of Mechanical and Aerospace Engineering, Faculty of Science and Engineering, Tokyo University of Science, Chiba, Japan

**Keywords:** Water sound, Subjective evaluation, Hypersonic effect, Ultrasonic sound, Electroencephalography, Physiological measurement, Auditory stimulus, Physiology, Psychology

## Abstract

This study aimed to investigate the effects of reproducing an ultrasonic sound above 20 kHz on the subjective impressions of water sounds using psychological and physiological information obtained by the semantic differential method and electroencephalography (EEG), respectively. The results indicated that the ultrasonic component affected the subjective impression of the water sounds. In addition, regarding the relationship between psychological and physiological aspects, a moderate correlation was confirmed between the EEG change rate and subjective impressions. However, no differences in characteristics were found between with and without the ultrasound component, suggesting that ultrasound does not directly affect the relationship between subjective impressions and EEG energy at the current stage. Furthermore, the correlations calculated for the left and right channels in the occipital region differed significantly, which suggests functional asymmetry for sound perception between the right and left hemispheres.

## Introduction

The effects of acoustic reproduction in a higher frequency over audible range on the psychophysiology of humans and animals have been investigated. Recently, it was reported that high-frequency acoustic reproduction can contribute to life-span extension in mice^1^. The improvement of anhedonia symptoms using high-frequency sound reproduction in humans has also been pointed out^2^. Regarding their effects on humans, in 2000, Ohashi et al.^3^ reported that gamelan music from Bali, which has frequency components above 22 kHz outside the audible range, affects human brain activity. That study revealed that sound stimuli with components outside the audible range were significantly activated by alpha-band electroencephalography (EEG). The psychological effects were also mentioned, and the results of subjective experiments on sound impressions using adjectives showed significant differences depending on the presence or absence of frequency components above the audible range. Subsequently, this so-called “hypersonic effect” was verified by the same research group, which found that the activation of brain wave is influenced by factors other than air-conducted hearing^4^, and that frequency components above 32 kHz have the effect of increasing the alpha-band EEG power, whereas those below 32 kHz have the inverse effect of decreasing the alpha-band EEG power^5^. Another research group replicated this result using classical music (J. S. Bach), reporting that listening to music with abundant high-frequency components above the audible range increased the alpha-band EEG power^6,7^. However, no differences in subjective impressions were found between the cases with and without high-frequency components.

Each of these previous studies had different views on the effects of exposure to sounds with high-frequency components that are basically inaudible via the air-conducted hearing path of humans. Muraoka et al.^8^ and Plenge et al.^9^ concluded that there is no conscious recognition of differences in sound quality, even when sounds in the frequency range of 15 kHz or higher are included. This finding has been used as original data for determining standards regarding the sampling frequencies for the digital CD and DAT formats. Kuribayashi et al.^6,7^ also found no difference in subjective impressions depending on the presence or absence of high-frequency components. In contrast, Oohashi et al.^3^ pointed out that the presence or absence of high-frequency components influences subjective impressions, but this conclusion was limited to cases in which gamelan sounds were used as the stimuli. Thus, whether psychological effects can be confirmed for other sound types, such as natural sounds, remains unknown. A previous study investigated the effects of water sounds on reducing stress^10^. More specifically, natural sounds such as river sounds are simple and may promote relaxation in humans^11^. It is interesting to see how frequency components outside the audible frequency range affect the psychophysiological aspects of humans. However, to the best of our knowledge, no studies have investigated the effects of inaudible sound components.

Given this background, the present study aimed to investigate the effects of an ultrasonic component with abundant high-frequency components above 20 kHz on the subjective impressions caused by a waterfall and a stream. Herein, considering the physiological characteristics of EEG, we investigate the mechanisms underlying the psychophysiological effects of acoustic exposure to broadband sounds outside the audible range.

## Results

### Effect of ultrasonic sound on psychological response

**Figure 1:**
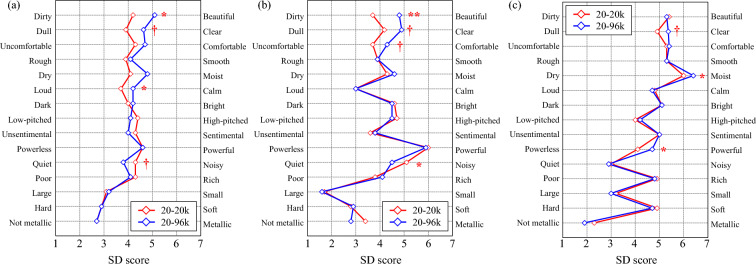
Average profile ratings obtained using the SD method for the sound stimuli of (**a**) WF_66_20k and WF_66_96k, (**b**) WF_76_20k and WF_76_96k, and (**c**) ST_66_20k and ST_66_96k.

The results of the subjective evaluation experiment using the semantic differential (SD) method are shown in Fig. 1 as a profile. The figure presents the average of the scores answered by each subject under three types of acoustic stimuli: WF_66_*, WF_76_*, and ST_66_*. Table 1 shows the results of a statistical test of the difference between the results with and without the reproduction of the ultrasonic component.

**Table 1:** Statistical results of the semantic differential scores for the conditions with and without ultrasonic components (†: *p* < 0.1, *: *p* < 0.05, **: *p* < 0.01). The effect size was calculated using Cohen’s *d*.

Auditory conditions			WF_66_**				WF_76_**				ST_66_**			
Domain			Mean ± SD (20 k vs. 96 k)	*p-*value	*d*	Sign.	Mean ± SD (20 k vs. 96 k)	*p-*value	*d*	Sign.	Mean ± SD (20 k vs. 96 k)	*p-*value	*d*	Sign.
Beautiful	–	Dirty	4.2 ± 1.3 < 5.1 ± 0.7	0.02	0.89	*	3.7 ± 1.6 < 4.8 ± 1.1	0.003	0.82	**	–			
Clear	–	Dull	3.9 ± 1.3 < 4.6 ± 0.94	0.07	0.70	**†**	4.2 ± 1.6 < 4.9 ± 1.6	0.05	0.31	*	4.9 ± 0.98 < 5.4 ± 0.9	0.09	0.53	**†**
Comfortable	–	Uncomfortable	–				3.7 ± 1.6 < 4.3 ± 1.5	0.08	0.31	**†**	–			
Smooth	-	Rough	–				–				–			
Moist	–	Dry	–				–				6.0 ± 0.89 < 6.4 ± 0.66	0.04	0.51	*
Calm	–	Loud	3.7 ± 1.2 < 4.2 ± 1.3	0.05	0.40	*	–				–			
Bright	–	Dark	–				–				–			
High-pitched	–	Low-pitched	–				–				–			
Sentimental	–	Unsentimental	–				–				–			
Powerful	–	Powerless	–				–				4.1 ± 1.2 < 4.7 ± 0.90	0.05	0.56	*
Noisy	–	Quiet	4.3 ± 0.9 > 3.8 ± 0.98	0.10	0.53	**†**	5.1 ± 1.1 > 4.5 ± 1.1	0.02	0.53	*	**-**			
Rich	–	Poor	–				–				–			
Small	–	Large	–				–				–			
Soft	–	Hard	–				**-**				–			
Metallic	–	Not metallic	–				–				–			

First, regarding the results for the waterfall sound reproduced at 66 dB (WF_66_*), differences in many adjectives were found between with and without the ultrasonic component (Fig. 1a). The adjective pairs that tended toward being different or significantly different were beautiful vs. dirty (*p* < 0.05, *d* = 0.89), clear vs. dull (*p* < 0.1, *d* = 0.70), calm vs. loud (*p* < 0.05, *d* = 0.40), and noisy vs. quiet (*p* < 0.1, *d* = 0.53). The waterfall sound with the ultrasonic component reproduced at 66 dB was more beautiful, clearer, calmer, and quieter than that without the ultrasonic component. As in the 66 dB case, the adjective pairs that showed significant differences for the waterfall sound played at 76 dB (WF_76_*) were beautiful vs. dirty (*p* < 0.005, *d* = 0.82), clear vs. dull (*p* < 0.05, *d* = 0.31), and calm vs. loud (*p* < 0.1, *d* = 0.31). In addition, a new trend toward a difference was found for comfortable vs. uncomfortable (Fig. 1b). Next, when the sound pressure levels (SPLs) were kept equal and the type of sound was a set to a stream, significant differences were found for moist vs. dry (*p* < 0.05, *d* = 0.51) and powerful vs. powerless (*p* < 0.05, *d* = 0.56), in addition to clear vs. dull (*p* < 0.1, *d* = 0.53), which was also observed under the other conditions (Fig. 1c). In most conditions, Cohen’s *d* ranged from medium to large.

Next, as shown in Table 1, differences were observed in perceptions related to the aesthetic aspects of sound, such as beauty, clarity, and comfort. Significant differences were also observed in adjectives related to the noisiness, loudness, and power of the sound. When the ultrasonic component was additionally reproduced, the sense of noisiness was reduced, whereas that of power was increased. Generally, the impression of the sound changed to more positive.

### Effect of ultrasonic sound on physiological response

**Figure 2:**
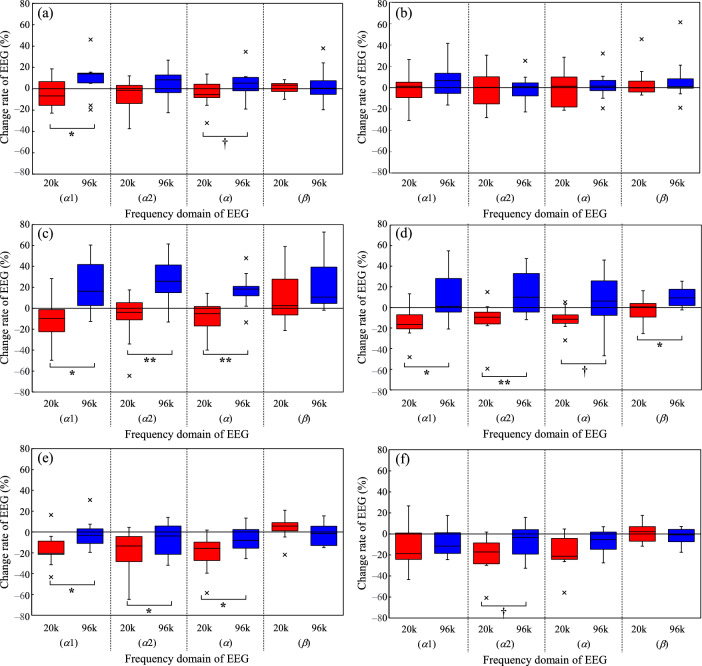
Results of a comparison of measured *α*1-, *α*2-, *α*-, and *β*-EEGs obtained from the (**a**, **c**, **e**) o1 and (**b**, **d**, **f**) o2 channels. The panels show the results for the sound stimuli of (**a**, **b**) WF_66_20k and WF_66_96k, (**c**, **d**) WF_76_20k and WF_76_96k, and (**e**, **f**) ST_66_20k and ST_66_96k. The error bars represent ± SD of each data set. †: *p* < 0.1, *: *p* < 0.05, **: *p* < 0.01.

The results for the EEG change rate in each frequency domain are shown in Fig. 2. Figure 2a, c, e and Fig. 2b, d, f show the results acquired in the o1 and o2 channels, respectively. Figure 2a–f show the results for WF_66_20k and WF_66_96k, WF_76_20k and WF_76_96k, and ST_66_20k and ST_66_96k, respectively.

**Figure 3:**
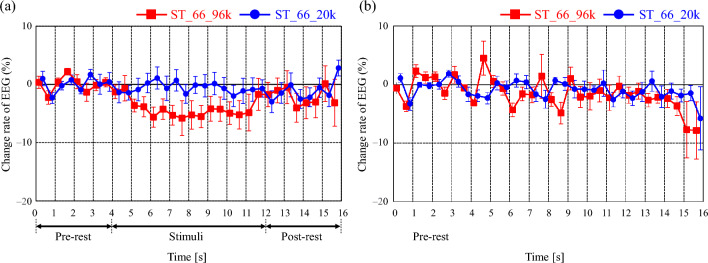
Example of measured time transient characteristics of *α*-EEGs obtained from the (**a**) o1 and (**b**) o2 channels. The results under the conditions of ST_66_20k and ST_66_96k are comparatively shown. The error bars represent ± SD of each data set.

An example of the EEG time series data used to obtain the change rate is shown in Fig. 3. Herein the results of measured time transient characteristics of *α*-EEGs obtained from the o1 and o2 channels under the conditions of ST_66_20k and ST_66_96k are comparatively shown. In this experiment, EEG waveforms were epoched in every 30 s, and each band component in each 30-s waveform was analyzed to determine the change ratio of the average power over 8 min of stimuli (EEG_Stim_) to the average power over 4 min of pre-rest (EEG_Base_) in each bands of *α*, *α*1, *α*2, and *β*, respectively. More detailed procedure is described in the following section of Physiological measurement. In the condition of o1 channel (Fig. 3a), *α*-EEG in the condition with the ultrasound component during stimulus playback is lower than that in the condition without the ultrasound component. On the other hand, in the o2 channel (Fig. 3b), there is no difference in the change rate during stimulation in both the condition with and without ultrasound. We integrated the power of the waveform for all time periods during the stimulation and obtained EEG_Stim_ to obtain a single index of the change rate. Then, as shown in Fig. 2e and f, the former figure shows a significant difference in the *α*-EEG band and the latter shows no significant difference. By applying this manipulation to the EEG results of the other conditions, the effects of EEG changes caused by each of the acoustic stimuli in each condition can be evaluated.

First, Fig. 2c, d shows that the *α*-EEG was increased by additionally reproducing the ultrasonic component in most bands and channels. In Fig. 2a, b, which was obtained by decreasing the SPL by 10 dB, a difference in the o1 channel is seen in the *α*1 and *α* bands, but not in the other bands. On the other hand, in Fig. 2e, f, which shows a condition where the sound type was changed to a stream sound, the result obtained for o1 shows differences in the *α*1, *α*2, and *α* bands, while that for o2 shows differences in the *α*2 band, although these differences only indicated a trend. In the right channel o2, a difference is observed in the o2 band, although this was only a trend toward a significant difference.

Thus, it can be confirmed that exposure to ultrasound mainly affects the *α*-EEG, which is comparable to the results of a previous study^1^. Furthermore, the results of this study suggest that the effect of ultrasound reproduction is greatly influenced by the SPL and type of sound. In contrast, we could not confirm any specific effect of the difference between the left and right o1 and o2 channels.

### Effects of ultrasonic sound and the relationship between physiological and psychological responses

Table 2 shows the correlation coefficients between the SD scores and EEG indices obtained under the conditions with and without ultrasound components. The conditions with correlation coefficients > 0.4, which indicates a medium or higher correlation, are shown in red^12^. Comparing the results obtained for the o1 and o2 channels, the latter showed a relatively higher correlation between SD scores and the EEG change rate under both conditions (with and without the ultrasound component).

**Table 2:** Results of calculated correlation coefficients between the SD scores and EEG change rates for *α*1-, *α*2-, *α*-, and *β*-EEGs obtained at the o1 and o2 channels.

Auditory conditions			20 Hz–20 kHz												20 Hz–96 kHz												
EEG channel			O1					O2							O1					O2							
Domain			*α*1		*α*2	*α*	*β*	*α*1		*α*2	*α*		*β*		*α*1		*α*2	*α*	*β*	*α*1		*α*2		*α*		*β*	
Beauty	-	Dirty	0.27		− 0.06	0.15	0.08	**0.66**	*	0.11	0.23		− 0.02		0.17		**0.47**	0.25	0.04	0.22		0.08		0.32		0.06	
Clear	−	Dull	0.38		0.20	0.23	0.05	**0.43**		**0.46**	− 0.17		**− 0.59**	*	0.19		0.12	0.05	-0.20	**0.59**	*	**0.51**		0.24	**†**	**-0.42**	
Comfortable	–	Uncomfortable	0.19		0.05	0.17	0.06	**0.61**	*	0.06	0.26		0.05		**0.50**	**†**	**0.40**	0.32	-0.32	0.17		0.16		0.31		0.17	
Smooth	−	Rough	0.38		**0.47**	**0.40**	0.27	**0.45**		**0.49**	− 0.05	**†**	**− 0.40**		0.26		0.34	− 0.23	− 0.29	0.16		0.03		0.15		0.18	
Moist	−	Dry	0.25		0.16	0.23	0.07	**0.49**	**†**	**0.44**	0.09		− 0.34		− 0.04		− 0.01	0.19	− 0.32	**0.50**	**†**	**0.43**		0.18		− 0.18	
Calm	−	Loud	0.02		− 0.05	− 0.07	0.12	0.34		0.11	0.03		− 0.12		− 0.14		− 0.21	− 0.11	0.10	0.38		0.18		− 0.17		− 0.27	
Bright	−	Dark	− 0.20		0.17	0.11	0.09	− 0.32		**− 0.52**	0.26	**†**	**0.47**	**†**	− 0.22		− 0.06	− 0.02	0.14	− 0.33		− 0.31		**0.41**		0.23	
High− pitched	−	Low-pitched	**0.42**		0.16	0.36	0.19	− 0.05		− 0.39	0.08		0.17		− 0.34		− 0.35	**− 0.44**	− 0.09	− 0.26		**− 0.45**		0.02		0.19	
Sentimental	−	Unsentimental	0.18		0.25	0.12	0.34	0.30		0.18	− 0.08		− 0.16		− 0.01		0.11	0.25	0.00	0.39		**0.56**		0.39	*	− 0.24	
Powerful	−	Powerless	− 0.14		− 0.10	− 0.30	0.18	**− 0.47**		− 0.15	− 0.24		− 0.03		0.25		− 0.12	− 0.05	0.11	0.23		− 0.24		**− 0.47**		− 0.37	
Noisy	−	Quiet	− 0.36		− 0.21	− 0.14	− 0.02	− 0.39		**− 0.50**	0.22	**†**	**0.53**		− 0.01		− 0.14	− 0.15	− 0.03	**− 0.48**	**†**	**− 0.48**		− 0.04	**†**	**0.62**	*
Rich	−	Poor	**0.68**	*	**0.44**	**0.46**	− 0.20	− 0.25		**0.47**	0.14		**− 0.44**		0.14		0.09	0.30	− 0.26	**0.43**		**0.42**		0.11		− 0.35	
Small	−	Large	− 0.08		− 0.03	− 0.05	− 0.10	**0.46**		0.30	0.09		− 0.22		0.00		− 0.12	0.01	0.28	0.37		**0.59**	*	**0.56**	*	0.00	
Soft	−	Hard	− 0.10		0.39	0.32	− 0.05	0.37		**0.52**	0.37	**†**	− 0.09		− 0.06		− 0.05	0.02	0.21	**0.49**		**0.55**		0.26	**†**	**− 0.46**	
Metallic	−	Not metallic	0.32		0.18	0.35	0.22	− 0.19		**− 0.55**	0.12	**†**	0.32		0.03		− 0.22	− 0.25	0.22	− 0.18		**− 0.54**		− 0.39	**†**	− 0.06	

Significant values are in [bold].

Next, the correlation coefficients between the *α*- and *β*-EEGs were inverted in most cases. For example, the SD scores for clear vs. dull were positively correlated with *α*-EEG, but negatively correlated with *β*-EEG. Conversely, in the case of noisy vs. quiet, SD scores were negatively correlated with *α*-EEG and positively correlated with *β*-EEG. More specifically, *α*-EEG and *β*-EEG seemed to be strongly associated with positive and negative emotions, respectively. For example, in a study of the built environment, *α*-EEG increased in a comfortable environment^13^, whereas *β*-EEG increased in a stressful environment^14^, suggesting that the correlation between *α*- and *β*-EEGs in the present study is based on the same mechanism.

## Discussion

First, the results from Fig. 1 and Table 1 suggest that the additionally reproduced ultrasonic component affects subjective impressions. Specifically, an effect was observed for the evaluation terms related to aesthetic and power impressions. In a previous study by Kuribayashi^5^, the first 200-s portion of French Suite No. 5 by J. S. Bach (on cembalo, 24-bit quantization, 192 kHz A/D sampling) was selected as the sound stimuli, and was reproduced by removing higher components using a low-pass finite impulse response digital filter with a very steep slope. As detailed acoustic spectral information on this acoustic signal is not available, it is not possible to compare directly the acoustic energy of the included ultrasonic waves between the above experiment and the present study. However, it is true that the water sound used in the present study has a continuously abundant signal from 20 to 96 kHz, which may have strongly influenced the results. In contrast, Oohashi et al.^1^ conducted a subjective evaluation experiment using gamelan music from Bali, which has a continuously decaying abundant component similar to the water sound in the ultrasonic domain, and reported finding significant differences (*p* < 0.01) in the following scales: soft vs. hard, reverberant vs. percussive, comfortable to ears vs. uncomfortable to ears, and rich in nuance vs. lacking in nuance. In that study, the acoustic stimuli were adjusted to a comfortable SPL for each of the subjects, where the maximum SPL distributed approximately 80–90 dB at the listening position. Those SPLs are about 10–20 dB higher than the SPLs used in the present study. In any case, comparing these results, it can be said that the time and frequency characteristics of the sound source signal influenced whether the ultrasonic component had affected subjective impressions. On the other hand, in the present study, even at a relatively low SPL of 66 dB, the ultrasonic components could affect subjective impressions depending on the type of sound source. However, further clarification of how subjective impressions are affected when the SPL is changed requires more detailed subjective evaluation experiments controlled at more finely categorized SPLs.

On the contrary, the present results also confirmed that the additionally reproduced ultrasonic components affect the EEG, as shown in Fig. 2. In particular, *α*-EEG was increased by the influence of the water sound, as shown in a previous study. As mentioned above, increasing the SPL of the waterfall increased the frequency of significant increases in *α*-EEG. However, this was dependent on the EEG change rate; thus, a more detailed verification is needed to determine the nature of this relationship.

Finally, the results in Table 2 indicated a significant correlation between SD scores and the EEG change rate. However, no significant difference between correlations was observed when ultrasound was or was not included. In other words, the ultrasound component increased *α*-EEG and improved the impression, but this effect did not have a significant impact on the relationship between subjective impressions and EEG. However, as mentioned above, the situation was very different for the left and right channels of o1 and o2 placed in the occipital region, with an increase in the number of pairs showing a moderate or higher correlation at o2. This is interesting from the viewpoint of the relationship between subjective impressions and physiological responses to a general water sound that contains more than a certain wide frequency band component, although it occurred regardless of the presence or absence of ultrasound reproduction. In a previous study, music perception was shown to be lateralized to the right hemisphere of the brain to a considerable extent^15^. A study target Indian music^16^ found that the right hemisphere is more adapted to emotional categorization for happiness and sadness, whereas another study^17^ questioned this concept of the lateralization of music perception. As the asymmetry suggested in the correspondence between subjective impressions of water sounds and the EEG change rate in the present study is also consistent with previous findings, the respective contributions to sound perception of the right and left hemispheres of the brain suggest a functional asymmetry. Furthermore, the asymmetric distribution of the EEG even for the water sounds, which do not directly affect human emotions like music, is a point that has not been observed in previous studies. On the other hand, the data obtained in this study did not allow us to extract fully how the relationship between subjective evaluations and EEG changes depend on the presence or absence of ultrasonic components. This will require further verification through experiments under more detailed control.

### Limitations

This study has following limitations. Fist, although the total number of the participants with 10 subjects was determined by following the previous study^18^, it is considered necessary to verify the findings of this study in an extended experiment with a larger sample size which can be determined based on the means and standard deviations obtained by this experiment. Second, the current study presented results focusing on the psychological and physiological effect of the ultrasonic components including quite broad frequency band from 20 to 96 kHz. Herein this study lacks controlled experimentation that isolates specific frequency bands in the higher frequency bands above 20 kHz. By adopting such a more detailed experimental design, more specific results can be obtained.

## Methods

### Experimental procedure

**Figure 4:**
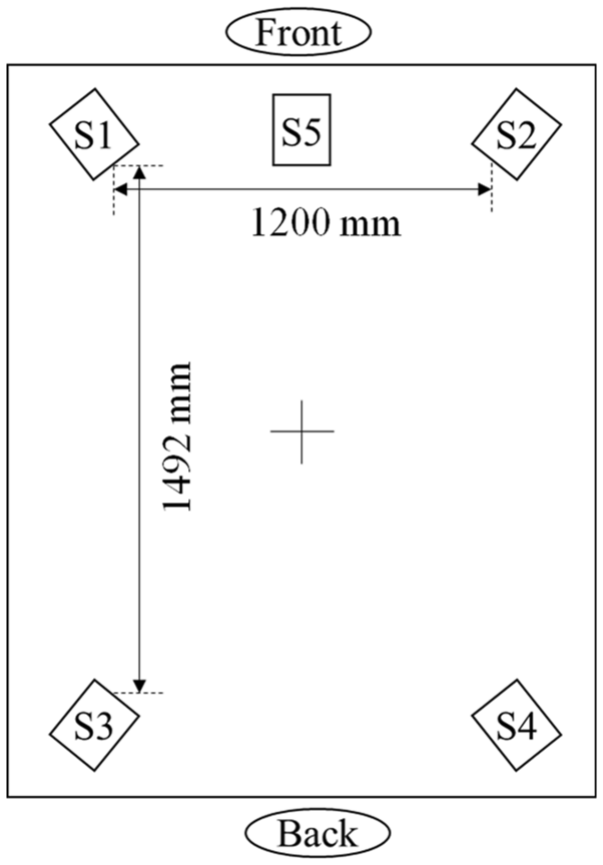
Spatial relationship between each of the speakers and the subject (+).

**Figure 5:**
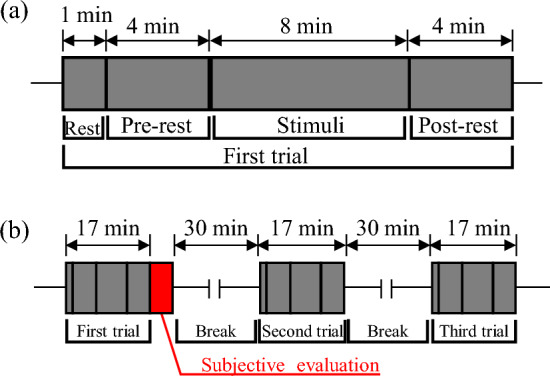
Experimental flow for (**a**) each of the acoustic conditions, and (**b**) the overall flow of experiment, including the three acoustic conditions.

The overall scheme of the experiment is described as follows. To ensure a quiet environment, the experiment was conducted in a soundproof room with an SPL difference of approximately 35 dB. The acoustic reverberation was suppressed by coating the surface inside the soundproof room by porous absorbers. The environment inside the room was kept dark to avoid the influence of visual stimuli, including the light environment, on the measured EEG. Related to this, the subjects' eyes were kept closed during all the experiments except for the rest time between each condition. As shown in Fig. 4, the subject was placed at the center of the soundproof room. Four loudspeakers (D-D2E; ONKYO, Osaka, Japan) and one woofer (HS8S; Yamaha, Hamamatsu, Japan) were set at each of the corners and in front of the subject, respectively, and the target sounds were reproduced. The experimental procedure used in the present investigation is shown in Fig. 5. A 1-min rest period was included, followed by a 4-min pre-rest period, which was used as a baseline for the EEG measurements. After that, 8 min of auditory stimulation were reproduced, followed by a post-rest period. Overall, the EEG response to each of the auditory stimuli was measured for 16 min. After that, subjective evaluation of the given acoustic condition was performed by the subject. Three types of acoustic auditory stimuli were used, as shown in Table 3: the sound of waterfalls with equivalent continuous A-weighted SPLs (*L*_Aeq_) of 66 dB (WF_66_20k and WF_66_96k) and 76 dB (WF_76_20k and WF_76_96k), and the sound of a river with *L*_Aeq_ of 66 dB (ST_66_20k and ST_66_96k). For these conditions, the sound files including the low-frequency components up to 20 kHz were also treated as stimuli, along with the original file with frequency components up to 96 kHz. Further details of the above conditions are described in section “Effects of ultrasonic sound and the relationship between physiological and psychological responses”.

**Table 3:** Experimental conditions of the adopted acoustic stimuli.

Type	Sound	*L*_Aeq_ [dB]	Frequency comp. [Hz]
WF_66_20k	Waterfall	66	20–20 k
WF_66_100k			20–96 k
WF_76_20k		76	20–20 k
WF_76_100k			20–96 k
ST_66_20k	Stream	66	20–20 k
ST_66_100k			20–96 k

### Participants

In total, 10 subjects (5 males and 5 females, mean age ± standard deviation: 21.9 ± 1.1 years) participated in the experiment. The number of subjects was determined with reference to a previous experiment^18^ in which an equivalent number of subjects was used to examine the relationship between subjective impressions of sound and its effects on neurologic behavior. All participants were recruited by an e-mail sent to students at the author’s institution. In accordance with EN 50332-1 and -2 proposed by the European Committee for Electrotechnical Standardization^19,20^ as sound pressure regulations for portable audio players and the ethical guidelines of Tokyo University of Science, this experimental study was conducted to be noninvasive. Informed consent for the experiment was obtained from all participants after being briefed on the purpose of the study and experimental methods, as well as the anonymization and use of data. Prior to the study, the participants were asked about their hearing ability, which was tested using the hearWHO hearing test app^21^, and assured that all of their ears were normal (all participants had a score > 75, indicating good hearing).

### Acoustic stimuli

In this study, naturally occurring sounds of streams and waterfalls were used as acoustic stimuli. It is significant to examine the psychophysiological effects of water sounds, which have a wide range of frequency components, because previous studies have examined the effects of water sounds^22^, mainly stream and waterfall sounds, to enhance the sound environment and to mask road traffic noise^23^.

The reproduced stream and waterfall sounds with frequency components up to 96 kHz were recorded at the Watarase River (Ashikaga city, Tochigi, Japan) and Otaki (Kanuma city, Tochigi, Japan), respectively, using two monaural ¼-inch microphones (378C01; PCB Piezotronics, Depew, NY, USA) and two monaural ½-inch microphones (378B02; PCB Piezotronics). As shown in Fig. 4, a five-channel speaker system including one channel with a woofer was used in this experiment. A two-way speaker with a crossover frequency of 2.5 kHz and a frequency response from 50 Hz up to 100 kHz (D-D2E; ONKYO, Osaka, Japan) was used as the four-channel loudspeaker; this speaker was capable of sufficiently driving the frequency range of the acoustic stimuli used in this study. Then, the sound recorded by the four microphones was reproduced through four speakers using two ¼-inch microphones with sensitivity up to 100 kHz and two ½-inch microphones with sensitivity up to 20 kHz. Although it is possible to use four ¼-inch microphones to record the sound for all channels, the small size of these microphones makes their self-noise more prominent than that of ½-inch microphones, and noise in the audible frequency range may cause problems in terms of subjective evaluations. Therefore, the sound recorded by the ¼-inch microphone was reproduced from the two front speakers to radiate the ultrasonic component to the subject efficiently, and the sound recorded by the ½-inch microphone was reproduced from the two rear speakers. The woofer in front of the subject reproduced the sound recorded by the ½-inch microphone. To reproduce the sounds, a PC was connected to an audio interface (UR44C; Steinberg, Hamburg, Germany), and the sound files were converted to analog signals and reproduced from the speakers. As the maximum sampling frequency of the audio interface is 192 kHz, the maximum upper limit frequency for recording and playback of the sound sources used in this study was set to 96 kHz.

The SPLs for each of the sound stimuli listed in Table 1 were set for the following reasons. First, the equivalent continuous A-weighted SPLs presented were 76 dB for the waterfall sound and 66 dB for the stream sound; these were the actually measured SPLs at the recording locations. To evaluate the differences in the presented SPLs, a waterfall sound stimulus with an equivalent continuous A-weighted SPL of 66 dB was additionally prepared by digitally attenuating the amplitude of the original sound wave file.

**Figure 6:**
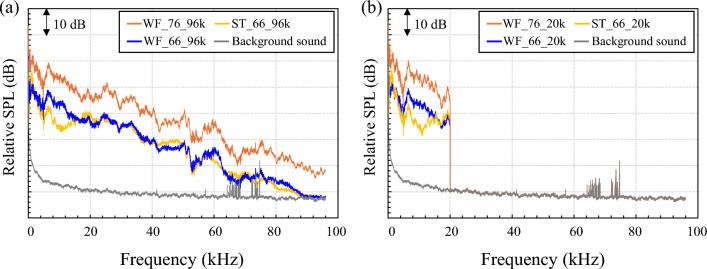
Frequency characteristics of the adopted water sounds for waterfalls and streams under condition (**a**) with an ultrasonic component and condition (**b**) without an ultrasonic component.

In this study, as already mentioned, two types of sounds were prepared for the various acoustic stimuli above, one with components up to 96 kHz as the original sound, and the other with components up to 20 kHz obtained by low-pass filtering. Then, the effects of the two kinds of stimuli on human psychophysiological states were compared and discussed. All of the above low-pass filtering and other types of digital processing were carried out using the Audition program (Adobe, San Jose, USA). The frequency characteristics of the various acoustic stimuli measured at the center of the subject’s head when each of the two sounds was reproduced are shown in Fig. 6. The three sounds shown in Fig. 6a contain an abundant frequency component > 20 kHz. By blocking these components, as shown in Fig. 6b, sound stimuli without ultrasonic components were generated and presented.

### Psychological measurements

**Figure 7:**
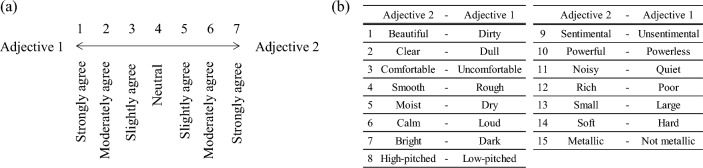
(**a**) The seven-point Likert scale used in the SD experiment and (**b**) the adjective pairs for positive (Adjective 2) and negative (Adjective 1) poles.

The subjective impressions of the simulated acoustic stimuli were measured by a subjective evaluation test using the SD method^24^. In this evaluation, a seven-point Likert scale (from “Strongly disagree” to “Strongly agree”) was used (Fig. 7a). Then, the 15 adjectives shown in Fig. 7b were used.

### Physiological measurements

**Figure 8:**
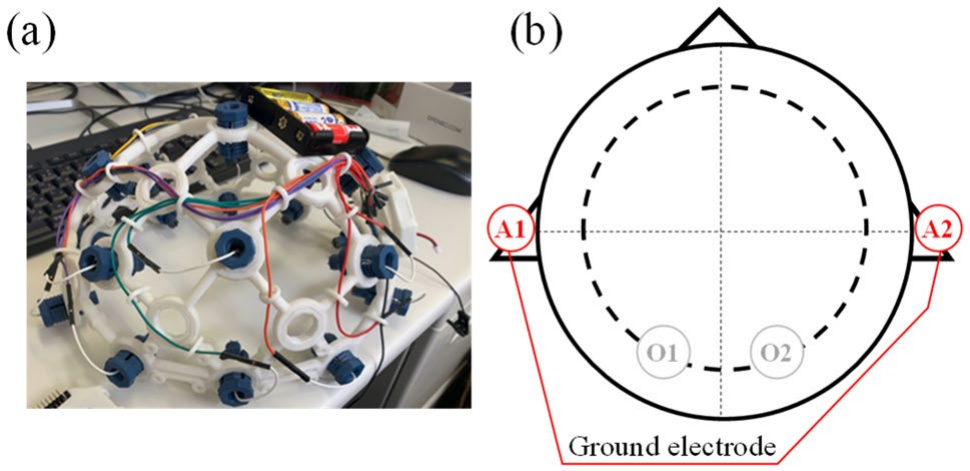
(**a**) EEG adopted in this study. (**b**) The measured channels for EEG were o1 and o2 on the posterior part of head, and A1 and A2 for the ground electrodes on the earlobes.

In this study, in addition to the psychological responses described in the previous section, EEG was observed to examine physiological responses in relation to psychological responses. EEG was measured using the Ultracortex Mark IV (OpenBCI, Brooklyn, NY, USA), as shown in Fig. 8a. The measured EEG waveforms were analyzed by using MATLAB (The Mathworks, Massachusetts, USA) with EEGLAB. The sampling rate of the measurements was 250 Hz. The measured waveform data were recorded via Bluetooth. As the main purpose of this study was to examine the subjective impressions caused by reproduced water sounds with ultrasound components in a relaxed situation, we decided to use as few channels as possible so as not to affect the natural impressions. Previous studies have shown that exposure to ultrasound components above the audible frequency range increases *α*-EEG^1^, and thus, changes in *α*-EEG were also expected in this study. The occipital significance for *α*-EEG^25,26^ have been reported, and changes in *α*-EEG at the occipital region have also been found in relation to comfort^27^. Therefore, the two measurement points shown in Fig. 8b (o1 and o2) were selected in this study. EEG recordings were derived using the monopolar derivation method with the earlobe as the reference electrode.

The EEG analysis methods are described as follows. First, the EEG indices in the respective frequency domains were obtained as follows. The low-frequency components were filtered out using a low-pass filter with frequency components up to 30 Hz. The unnecessary pulsive waves of artifacts caused by eye movements were manually excluded. Next, the waveform was cut into multiple 10-s segments, and band-limited powers of *PSD*(*f*) from *f*_1_ Hz to *f*_2_ Hz in the α1 domain from 8 to 10 Hz, α2 domain from 10 to 13 Hz, overall α domain from 8 to 13 Hz, and β domain from 13 to 30 Hz were calculated as follows:

1 $$P_{{f_{1} - f_{2} }} = \mathop \smallint \limits_{{f_{1} }}^{{f_{2} }} PSD\left( f \right)df$$

where *PSD*(*f*) is the power spectrum density obtained from the fast Fourier transform treatment of each segment. Physiological indices may have different baseline physiological quantities depending on the physiological and psychological state at the time of their measurement. Therefore, it is difficult to compare absolute values of indices among subjects. So, as shown in Fig. 5a, a pre-rest period was set during each measurement, and the physiological parameters measured during that period was used as the baseline value (EEG_Base_). Then, the ratio of the physiological parameters when acoustic stimulation was applied (EEG_Stim_) to this baseline value was calculated as the change rate and discussed. The EEG change rate in each frequency domain *R* was calculated as follows:

2 $$R = \frac{{EEG_{{{\text{stim}}}} - EEG_{{{\text{base}}}} }}{{EEG_{{{\text{base}}}} }} \cdot 100$$

Regarding the frequency domain segmentation of the EEG, the *α* band, which has been found to differ significantly in a previous study^1^, was mainly discussed. The *β* band, which is adjacent to the *α* band, was additionally treated. To discuss further details of the *α* band, the segmentation method of the *α* band into *α*1 and *α*2 was also adopted, in accordance with a previous study on the hypersonic effect^5^. Finally, the *α*-EEG, *α*1-EEG, *α*2-EEG, and *β*-EEG change rates were treated.

### Statistical analysis

Statistical analysis was performed using BellCurve for Excel (Social Survey Research Information Co., Ltd., Tokyo, Japan). The difference between the with and without ultrasound component conditions was tested using a two-tailed Student’s *t*-test. For all analyses, *p* values < 0.05 were considered significant. To clarify the psychological mechanisms of highly variable events regarding the residents’ impressions of practical audiovisual stimuli, such as the urban environment, *p* values between 0.1 and 0.05 were discussed together as indicating a trend toward significance. The parameters of Cohen’s *d* were calculated to estimate effective sizes^28^, where the threshold of the extent of effectiveness is defined as follows: 0.2 < *d* < 0.5: small, 0.5 < *d* < 0.8: medium, and 0.8 < *d* < 1.0: large.

## Data Availability

The datasets used and/or analyzed during the present study are available from the corresponding author upon reasonable request.
